# The Role of Zooplankton Community Composition in Fecal Pellet Carbon Production in the York River Estuary, Chesapeake Bay

**DOI:** 10.1007/s12237-024-01442-8

**Published:** 2024-11-13

**Authors:** Kristen N. Sharpe, Deborah K. Steinberg, Karen Stamieszkin

**Affiliations:** 1 Virginia Institute of Marine Science, 1375 Greate Rd., Gloucester Point, VA 23062 USA; 2 Bigelow Laboratory for Ocean Sciences, 60 Bigelow Dr., East Boothbay, ME 04544 USA

**Keywords:** Copepod, Diel vertical migration, *Acartia*, Particle export, Biological pump

## Abstract

Zooplankton play a key role in the cycling of carbon in aquatic ecosystems, yet their production of carbon-rich fecal pellets, which sink to depth and can fuel benthic community metabolism, is rarely quantified in estuaries. We measured fecal pellet carbon (FPC) production by the whole near-surface mesozooplankton community in the York River sub-estuary of Chesapeake Bay. Zooplankton biomass and taxonomic composition were measured with monthly paired day/night net tows. Live animal experiments were used to quantify FPC production rates of the whole community and dominant individual taxa. Zooplankton biomass increased in surface waters at night (2- to 29-fold) due to diel vertical migration, especially by *Acartia* spp. copepods. Biomass and diversity were seasonally low in the winter and high in the summer and often dominated by *Acartia* copepods. Whole community FPC production rates were higher (3- to 65-fold) at night than during the day, with the 0.5–1 mm size class contributing 2–26% to FPC production in the day versus 40–70% at night. An increase in the relative contribution of larger size fractions to total FPC production occurred at night due to diel vertical migration of larger animals into surface waters. Community FPC production was highest in fall due to increased diversity and abundance of larger animals producing larger fecal pellets, and lowest in summer likely due to top-down control of abundant crustacean taxa by gelatinous predators. This study indicates that zooplankton FPC production in estuaries can surpass that in oceanic systems and suggests that fecal pellet export is important in benthic-pelagic coupling in estuaries.

**Supplementary Information:**

The online version contains supplementary material available at 10.1007/s12237-024-01442-8.

## Introduction

Zooplankton play a key role in the ocean’s biological pump — the biologically mediated transport of surface community production (i.e., fixed carbon) to depth — by ingesting particulate organic carbon (POC) mostly in the form of phytoplankton in surface waters and producing sinking fecal pellets (Steinberg and Landry 2017). Zooplankton fecal pellets passively sinking from surface waters can comprise a large proportion of POC flux to depth (Turner 2015). Sinking POC, as fecal pellets or in other forms, that is not consumed and remineralized in the water column is deposited onto the ocean floor where it can support benthic communities or be buried and effectively sequestered. In estuaries, partially enclosed coastal waterways where freshwater from rivers and streams mixes with seawater from the coastal ocean, POC can also be exported out of the estuary through tidal flushing (Pinckney et al. 2001). Cross-system analyses of POC input and respiration in estuaries indicate that one-quarter of primary production and organic carbon inputs on the surface is respired at the bottom (Nixon 1981). In contrast, in the open ocean globally, only ~ 0.1% of surface carbon is deposited onto the seafloor on a time scale of millennia (Berelson 2001). Still, the biological pump and associated carbon exporting processes set up the vertical gradient of dissolved inorganic carbon in surface waters, enhancing ocean uptake of atmospheric CO_2_ (Siegel et al. 2023). Zooplankton-mediated export thus can play a significant role in the global carbon budget and ultimately in climate regulation.

The structure of the zooplankton community affects particle export and leads to differential carbon flux rates in different regions of the ocean (Wilson et al. 2008; Stukel et al. 2013; Dagg et al. 2014; Steinberg and Landry 2017; Steinberg et al. 2023). Different zooplankton taxa create morphologically distinct fecal pellets with variable carbon content and sinking rates (Turner 2015; Stamieszkin et al. 2021) and the likelihood of remineralization versus burial. Zooplankton species composition varies on seasonal (and interannual) time scales because of changing environmental variables and food availability, leading to seasonal variation in vertical carbon export (Riser et al. 2010). Zooplankton species composition also varies between day and night due to diel vertical migration, which can affect particle flux. Diel changes in surface zooplankton community composition in the northeast Pacific Ocean, for example, led to fecal pellet production rates that were on average double in surface waters at night compared to the day (Stamieszkin et al. 2021). Zooplankton that undergo diel vertical migration also play a role in the biological pump, actively transporting carbon to depth by grazing in the surface waters at night and migrating to deeper waters where they reside during the day and metabolize surface-ingested POC (Steinberg and Landry 2017). Studies of diel vertical migration have mostly focused on the open ocean (reviewed in Dawidowicz and Pijanowska 2018) although several have examined estuaries (Kimmerer et al. 2002; Naylor 2006; Chew et al. 2015; Vineetha et al. 2015). Only two prior studies of zooplankton diel vertical migration exist for Chesapeake Bay (Bosch and Taylor 1973; Cuker and Watson 2002).

Zooplankton fecal pellet production has only been measured in a few estuaries: the Krka Estuary in the Eastern Adriatic Sea (Svensen et al. 2007), the Yangtze Estuary in the East China Sea (Guo and Sun 2018), and the Chesapeake Bay (Saba et al. 2011; Stone and Steinberg 2018). These studies focused on fecal pellet production rates of dominant species only (e.g., copepods, gelatinous zooplankton) and suggest that rates vary on a seasonal basis as well as with estuarine physical and chemical conditions such as circulation, salinity, stratification, and nutrient inputs. Whole community-level fecal pellet production experiments are rare and include studies in Norway and the Antarctic polar front (Urban-Rich 2001), Monterey Bay and coastal California (Dagg et al. 2014), and the subarctic Northeast Pacific Ocean (Stamieszkin et al. 2021), with none in estuaries. However limited, data from prior studies suggest that rates of fecal pellet production and potential flux in estuaries can rival or surpass rates of fecal pellet export in oceanic systems which have received considerably more attention in this regard. This highlights the need for zooplankton fecal pellet production studies in estuaries where, compared to oceanic systems, the diversity of zooplankton is relatively low, but biomass is high (Park and Marshall 2000) due to high nutrient availability and associated primary production.

The objective of this study was to quantify diel and seasonal changes in zooplankton community composition and associated effects on fecal pellet carbon (FPC) production in the York River, a major tributary to the Chesapeake Bay, one of the world’s largest estuaries. The York River is characterized by complex physical, chemical, and biological interactions that cause broad variability on a diel, seasonal, and interannual basis, influencing the zooplankton community through altering the physical environment as well as the species composition of phytoplankton and other prey (Roman et al. 2001; Ludsin et al. 2009). We quantified both size-fractionated whole community-level and taxon-specific production rates of fecal pellets by zooplankton using experiments. This study represents the first in an estuary to quantify the diel and seasonal production of fecal pellets by the whole zooplankton community, which can be used both to inform the role of zooplankton in estuarine vertical carbon flux and for estuarine carbon cycle modeling.

## Methods

### Zooplankton Collection and Water Quality Monitoring

**Fig. 1:**
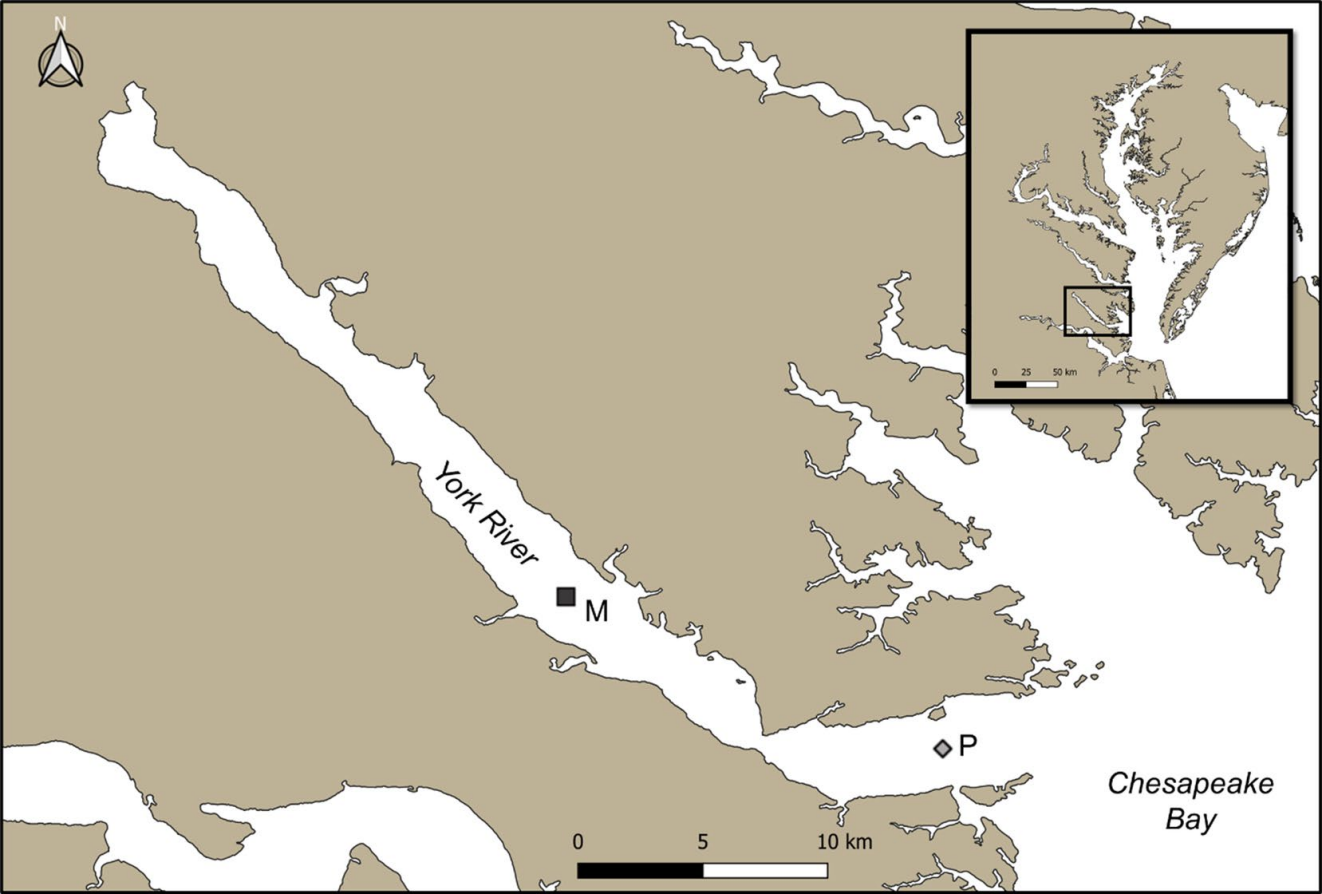
Sampling sites in the York River, Chesapeake Bay. Upriver mesohaline site (M; depth = 10.4 m; denoted by black square) and downriver polyhaline site (P; depth = 16.8 m; gray diamond). Location of the York River within the Chesapeake Bay region shown in the inset map

Meso- and macrozooplankton (zooplankton ≥ 200 µm in size) were collected from one mesohaline (37.3224°N, − 76.5997°W; depth = 10.4 m) and one polyhaline site (37.2371°N, − 76.4019°W; depth = 16.8 m) in the York River estuary (Fig. 1) from June 2019 to November 2020. Paired day/night sampling occurred approximately monthly in the polyhaline site, and once in each of four seasons in the mesohaline site. Diel sampling to quantify changes in zooplankton community structure due to diel vertical migration occurred ~ 12 h apart, and consistently during the early flood period of the tidal cycle. This period of the tidal cycle was chosen based on a prior study that recorded the highest surface mesozooplankton abundance during this phase in a similar shallow, temperate estuary (Chazarreta et al. 2015). Zooplankton were collected from net tows within the top 2 m of the water column based on previous work in Chesapeake Bay indicating diel migrators were concentrated in near sub-surface waters at night (Cuker and Watson 2002). All boat lights were turned off while towing at the collection site at night.

Zooplankton used for community structure and biomass measurements were collected using a 1-m diameter ring net (200 µm mesh) towed horizontally, using an electric winch, off the side of the vessel for 2–5 min. One such tow was performed on each cruise, and a General Oceanics mechanical flowmeter was used to measure the volume of water filtered through the net. The zooplankton net sample was immediately split on board, with half poured through nested sieves of 5-, 2-, 1-, 0.5-, and 0.2-mm mesh to produce five size fractions (0.2–0.5 mm, 0.5–1 mm, 1–2 mm, 2–5 mm, and > 5 mm). Each size fraction was rinsed onto pre-weighed 200 µm Nitex disks for biomass measurements. The remaining half-split of the tow was preserved in 4% buffered formaldehyde for later taxonomic identification and enumeration.

Three additional horizontal surface tows with a 1-m diameter ring net (one each with 200 µm, 500 µm, and 1600 µm mesh) were performed to collect live animals to be used in fecal pellet production experiments. The different sizes of mesh were used to exclude smaller animals in the larger animal size fractions in experiments, and a non-filtering cod end was used to maintain animals in good condition. Live animals were gently released into 20-l containers filled with whole, unfiltered seawater collected from the same location, and transported to shore for experiments.

During each cruise, water temperature, salinity, and pH were measured in the surface water using an Apera SX823-B pH/mV/conductivity meter. Light intensity was recorded at both the surface and ~ 0.5 m below the surface (Milwaukee MW700 LUX light meter). Water samples for chlorophyll-a analysis were collected in triplicate from just below the surface, filtered onto Whatman glass microfiber filters, extracted using buffered acetone in the dark for 24 h (Shoaf and Lium 1976), and analyzed using a 10 AU Turner Design fluorometer. Chlorophyll-a was used as a proxy for food availability for zooplankton in the fecal pellet production experiments.

### Zooplankton Size-Fractionated Biomass Analysis

Biomass filters were placed in a cooler with an ice pack to keep them cold for transport back to the lab, where they were then placed in a − 20 °C freezer. For processing, frozen biomass filters were thawed for at least 30 min and weighed on a microscale (Sartorius BP211D) to obtain wet weight. Filters were then dried for 24 h in a drying oven at 60 °C, removed, and weighed again to obtain dry weight. Dry weight biomass per cubic meter of each of the five size classes was calculated by dividing biomass measured by the volume of water filtered by the net (mg dry weight m^−3^).

### Zooplankton Taxonomic Analysis

Taxonomic identification of zooplankton was performed on preserved samples corresponding to days in which community-level fecal pellet production experiments occurred. The sample was first size-fractionated through a 1-mm sieve, with all animals > 1 mm identified to major taxon and enumerated. The < 1 mm size fraction was diluted to 50–100 × the total biovolume of animals present, and a 5-mL Stempel pipette was used to collect a subsample, ensuring a minimum of 100 non-*Acartia* spp. (an abundant calanoid copepod) animals were identified and enumerated. Once the identification of non-*Acartia* animals was complete, the < 1 mm size fraction was further diluted to 250–500 × the original biovolume of animals present, and a 5-mL Stempel pipette was used to collect a subsample for identification and enumeration of *Acartia* spp. copepods. A list of major taxa identified can be found in Table S1. Identifications were performed using an Olympus SZX10 stereo microscope at 70–250 × magnification.

### Fecal Pellet Production Experiment Setup

Both taxon-specific and community-level fecal pellet production experiments were performed, following the methods of Stamieszkin et al. (2021). All experiments were conducted on shore at in situ water temperature and light conditions present at ~ 0.5–1 m depth using a flow-through incubator covered with a light-filtering screen. For taxon-specific fecal pellet production experiments, relatively abundant species collected in each location (including *Acartia* spp. copepods, *Neomysis americana* mysids, and *Livoneca* sp. isopods) were sorted from diluted live tow samples by eye, or under a dissecting microscope, counted, and using a wide-bore pipette or spoon gently added to 1-l fecal pellet production experimental jars, designed for single-species experiments (Stamieszkin et al. 2021). These jars were fitted with an inset mesh-bottomed container within which animals were kept separated from their fecal pellets that sank through the mesh and concentrated at the bottom of the jar. Fecal pellet production from each species was measured with a minimum of 3 replicates, alongside 3 controls containing whole surface seawater only. At the end of the 4–6-h incubation, the inset containers were lifted out, and animals were either collected with forceps into small centrifuge tubes (mysids and isopods) or rinsed into small petri dishes (*Acartia* spp. copepods) and enumerated under a microscope before being frozen at − 20 °C. The contents of the outer container were poured through a 56-µm sieve to collect fecal pellets, which were then rinsed into separate centrifuge tubes and frozen at − 20 °C. For processing, thawed animals from experiments were filtered onto pre-weighed 0.2-mm Nitex disks to be used for dry-weight biomass measurements. Centrifuge tubes containing fecal pellets were thawed, all pellets were photographed and measured (see “Fecal Pellet Imaging and Carbon to Volume Conversion”) then concentrated onto glass fiber filters (GFFs) and frozen at − 20 °C awaiting analysis (see “Fecal Pellet Elemental Analysis”).

For community-level experiments, subsamples of the live animal collection tows were gently size-fractionated into 5 size classes using mesh-bottom containers (0.2–0.5 mm, 0.5–1 mm, 1–2 mm, 2–5 mm, > 5 mm; corresponding to size-fractionated biomass). As in taxon-specific experiments, these containers were also set into an outer container, keeping zooplankton above the mesh, and separated from their fecal pellets which were collected in the outer container (see Fig. 2 in Stamieszkin et al. 2021). Each community fecal pellet production experiment consisted of 12, 4-l containers: 2 replicates containing animals in each of the 5 size classes in unfiltered surface seawater, and 2 replicate controls containing unfiltered surface seawater only. The experimental containers were incubated for 4–6 h, after which the inset mesh-bottom containers were lifted out — removing the animals — which were rinsed into 15-mL centrifuge tubes and frozen at − 20 °C. Water and fecal pellets from the outer container were then poured through a 56-µm sieve to collect the fecal pellets, which were rinsed into separate centrifuge tubes and frozen at − 20 °C. Zooplankton and fecal pellet samples were processed similarly as with the community-level experiments.

### Fecal Pellet Imaging and Carbon-to-Volume Conversion

A calibrated microscope camera system (Olympus SZX 10 stereo dissecting microscope at 25 × magnification) was used to first image all fecal pellets used in elemental (CHN) analysis. The thawed pellet fractions were poured into a small, gridded petri dish and photographed. Measurements (using CellSens software) of pellet length and width were used to calculate fecal pellet volume (assuming a spheroid shape), and FPC content was measured using CHN analysis (see below). The measured carbon-to-volume relationship (*R*^2^ = 0.73) was used to calculate pellet carbon content when there was insufficient weight of pellets for a given replicate to detect pellet carbon using CHN analysis (Stamieszkin et al. 2021) (used for ~ 8% of samples; 17 of 225).

### Fecal Pellet Elemental Analysis

After imaging, zooplankton fecal pellets from experiments were filtered onto 25 mm combusted GFFs and then placed in a drying oven at 60 °C for a minimum of 24 h. The filters were then acidifed by fuming with HCl in a desiccator for at least 16 h to remove inorganic carbon (Gleiber et al. 2012) and again placed in the drying oven for an additional 24 h before being wrapped in 30-mm tin disks and pelletized. Once pelletized, samples were held in the drying oven at 60 °C until they were processed for organic carbon content. CHN analysis was performed using a Costech 4010 Elemental Combustion System.

### Calculation of Fecal Pellet Production Rate

Taxon-specific FPC production rates were calculated by subtracting the average carbon contained in control containers from each experimental replicate, which was then divided by the number of individuals in the replicate as well as the duration of the experiment to calculate a per-individual rate of FPC production (µg C ind^−1^ h^−1^) (Stamieszkin et al. 2021). Similarly, for whole community size-fractionated experiments, the average carbon contained in control containers was subtracted from each experimental replicate (each of the size-class containers was treated as a replicate). Mean fecal pellet production rates were calculated using the carbon content of pellets produced per dry weight of organisms in each of the five size fractions (mg C mgDW^−1^ h^−1^) and were averaged between replicates (*n* = 2) within each experiment. Calculated fecal pellet production rates were applied to measurements of size-fractionated mesozooplankton biomass (mg DW m^−3^) and summed to determine whole community fecal pellet production rates (mg C m^−3^ h^−1^) (Stamieszkin et al. 2021).

### Data Analysis

To statistically test differences in FPC production rates (i.e., diel, between taxa, or size classes), we first determined whether our data were normally distributed using visual normality plots (QQ plots and histograms). We used a paired sample *t*-test to compare taxa-specific fecal pellet production rates between the day and night and a one-way analysis of variance (ANOVA) to compare fecal pellet production rates between taxa. For community-level experiments, a one-way ANOVA was used to compare weight-specific FPC production rates and volume-specific FPC content between the two sampling sites, between the different size classes, and between day and night.

## Results

### Environmental Setting

Surface water temperatures were similar between the two sites, ranging from 8.3 to 30 °C at the polyhaline site (bottom depth = 16.8 m) and 9.3–30.4 °C at the mesohaline site (bottom depth = 10.4 m), and were lowest in February and at maximum in July (Supplementary Material Fig. S1a). Surface water salinity was typically 5 units lower in the mesohaline site than the polyhaline site and ranged from 5 to 19 (mesohaline) and 11 to 24 (polyhaline) (Supplementary Material Fig. S1b). Salinity dropped to a minimum at both sites in November 2020 due to a sustained, 3-day heavy rain event prior to sampling. Chlorophyll-a concentration varied considerably at both sites, with a range of 2.2–16.9 µg L^−1^ in the polyhaline site (highest in spring and summer and lowest in late fall) and 3.9–20.5 µg L^−1^ in the mesohaline site (highest in summer and generally lower in early spring and late fall). Overall, chlorophyll-a concentration was usually higher in the mesohaline site (Supplementary Material Fig. S1c).

### Diel and Seasonal Trends in Zooplankton Biomass and Size Structure

**Fig. 2:**
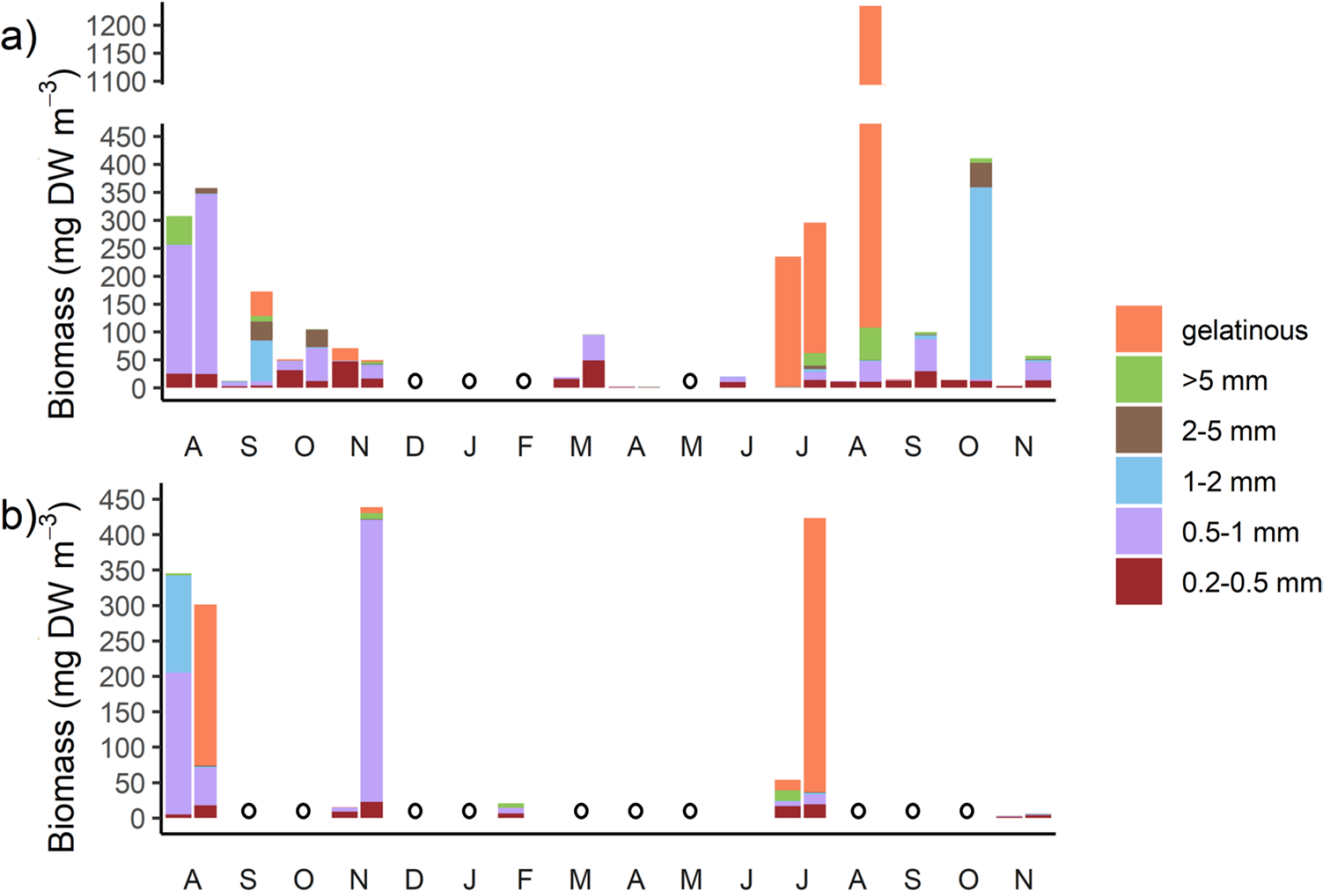
Size-fractionated mesozooplankton biomass in the polyhaline (**a**) and mesohaline (**b**) York River. For months with paired day-night tows, the first bar in the pair is day, and the second is night. Only day tows were performed in other months, with circles denoting months in which no sampling occurred. Gelatinous zooplankton (comprised of ctenophores, hydromedusae, and scyphomedusae; see Supplementary Material Table S1 and Supplementary Material Fig. S5) are shown as a separate category to better illustrate seasonal trends (note all are > 5 mm). For all tows *n* = 1

At the polyhaline site, zooplankton biomass increased in the surface waters at night 2- to 29-fold due to diel vertical migration, except for Nov. 2019, when biomass decreased in the surface waters at night (Fig. 2a). The size structure of the community also shifted from day to night, with an increase in biomass of intermediate and larger size classes (0.5–1 mm, 1–2 mm, and 2–5 mm) in surface waters at night. Total zooplankton community biomass was lowest in April and increased to maximum values in August of each year (Fig. 2a). Gelatinous zooplankton biomass was high and peaked in the summer months in 2020 due to the presence of large scyphozoan medusae but was lower in the summer of 2019 (Fig. 2a). At the mesohaline site, biomass — especially of the intermediate and large size classes — also generally increased in the surface waters at night, except for Aug. 2019 (Fig. 2b). Total community biomass was comparable to the polyhaline site (although mesohaline sampling was more limited), and gelatinous zooplankton biomass was high in summer of both 2019 and 2020 (Fig. 2b).

### Diel and Seasonal Trends in Zooplankton Abundance and Community Composition

**Fig. 3:**
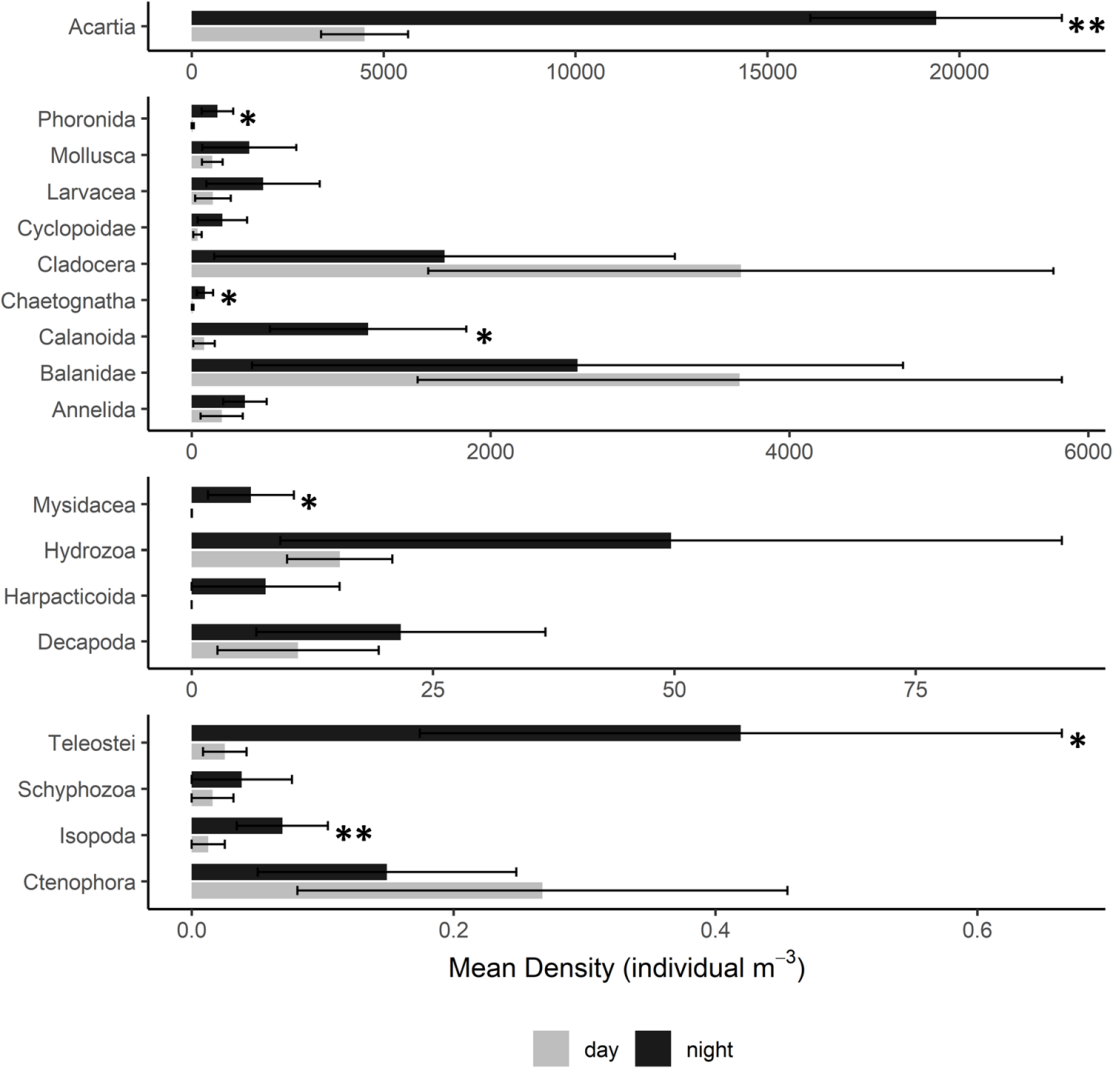
Zooplankton diel vertical migration in the polyhaline York River. Mean day and night density of major taxa in surface waters in the polyhaline sampling site, calculated across the time series (Nov. 2019 to Nov. 2020). Note different *x*-axis scales. **Taxa exhibiting the strongest patterns of diel vertical migration with higher mean densities at night than during the day (*p* < 0.05; student’s paired *t*-test) *Additional taxa exhibiting strong diel vertical migration behavior (0.05 < *p* < 0.10). *n* = 2–5 for day and night. Error bars = 1 standard error. (See Supplementary Material Table S1 for the full list and explanation of major taxonomic categories.)

Of the 18 major zooplankton taxa identified in the samples, 15 had higher mean density in surface waters at night than during the day, with two groups (*Acartia* spp. copepods and isopods) exhibiting the strongest patterns of diel vertical migration (*p* = 0.005 and *p* = 0.057, respectively; paired sample *t*-test, df = 5) and five — phoronids, chaetognaths, other calanoid copepods, mysids, and larval fishes — also exhibiting strong (*p* = 0.084, 0.086, 0.089, 0.102, and 0.087, respectively; paired sample *t*-test, df = 4) diel vertical migration patterns (Fig. 3). Three taxa (barnacle larvae-Balanidae, cladocera, and ctenophores) had lower mean density in the surface waters at night than during the day (Fig. 3). Among the taxa with higher mean density at night than during the day, mean night to day (N:D) abundance ratios ranged from 1.75 for decapods to 44.5 for chaetognaths. In addition to chaetognaths, the strongest vertical migrators included teleosts (mean N:D = 13.6), non-*Acartia* spp. calanoid copepods (11.9), annelids (9.64), and phoronids (8.62). N:D ratios could not be calculated for harpacticoid copepods, mysids, or scyphozoans, as they were either only present at night (harpacticoids and mysids) or otherwise paired monthly day and night abundance data was unavailable (scyphozoans).

**Fig. 4:**
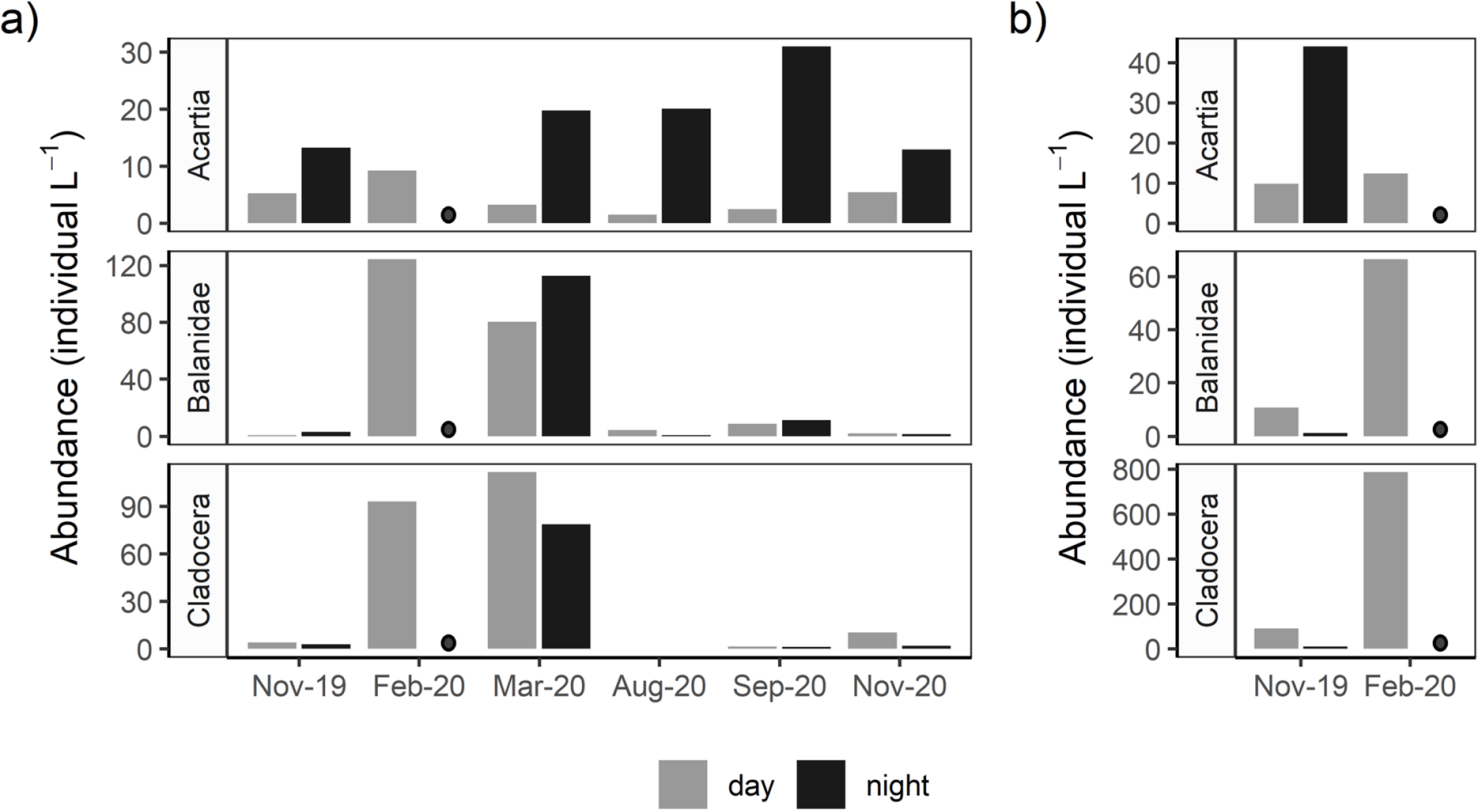
Monthly densities of *Acartia* spp. copepods and other abundant taxa of crustacean zooplankton in the polyhaline (**a**) and mesohaline (**b**) York River during the day and night. The dark circle in February 2020 denotes no night sampling performed, to distinguish from the absence of taxa in other months. (See Supplementary Materials Table S1 for the full list and explanation of major taxonomic categories.) For all tows *n* = 1

*Acartia* spp. copepods were the most abundant taxon in both sites, with densities of 1.5–31 individuals L^−1^ in the polyhaline and 10–44 individuals L^−1^ in the mesohaline site (Fig. 4a, b, respectively). In the polyhaline site, densities of most copepod groups (including *Acartia* spp., non-*Acartia* calanoids, and cyclopoids) were highest in Sept. 2020 (Fig. 4a, Supplementary Material Fig. S2a). The next most abundant taxa at both sites were cladocera (*Podon polyphemoides* and *Evadne nordmanii*) and barnacle (*Balanus* sp.) nauplii and cyprids, all which were most abundant in Feb. and Mar. 2020 in both the polyhaline and mesohaline sites (Fig. 4a, b) and occurred in low densities the rest of the year. Mysid and isopod densities at both sites were highest in the summer and fall at night, and decapod (primarily crab zoea) density was higher in the polyhaline site, with a peak in summer (Supplementary Material Fig. S3). Mollusks, chaetognaths, and annelids occurred in both sites, with mollusks and chaetognaths being most abundant in the summer and fall and annelids in the spring through summer (Supplementary Material Fig. S4). Other taxa found only in the polyhaline site included phoronid (horseshoe worm) larvae, larvaceans, and larval fishes — which were most abundant in the summer and fall at night.

The most abundant gelatinous zooplankton in both sites were hydrozoan medusae, primarily *Nemopsis bachei*, which were present throughout the spring and summer (Supplementary Material Fig. S5). Ctenophores (*Beroë ovata* and *Mnemiopsis leidyi*) were most abundant in both sites in Nov. 2019, but also in late summer 2020 in the polyhaline site (Supplementary Material Fig. S5a). Scyphozoan medusae (including *Chrysaora chesapeakei*, *Cyanea capillata*, and *Aurelia aurita*) occurred in Nov. 2019 (day) and Aug. 2020 (night) in the polyhaline site and in Feb. 2020 (day) in the mesohaline site (Supplementary Material Fig. S5a, b, respectively), which caused peaks in gelatinous zooplankton biomass (Fig. 2).

### Taxon-Specific Fecal Pellet Production Experiments

**Fig. 5:**
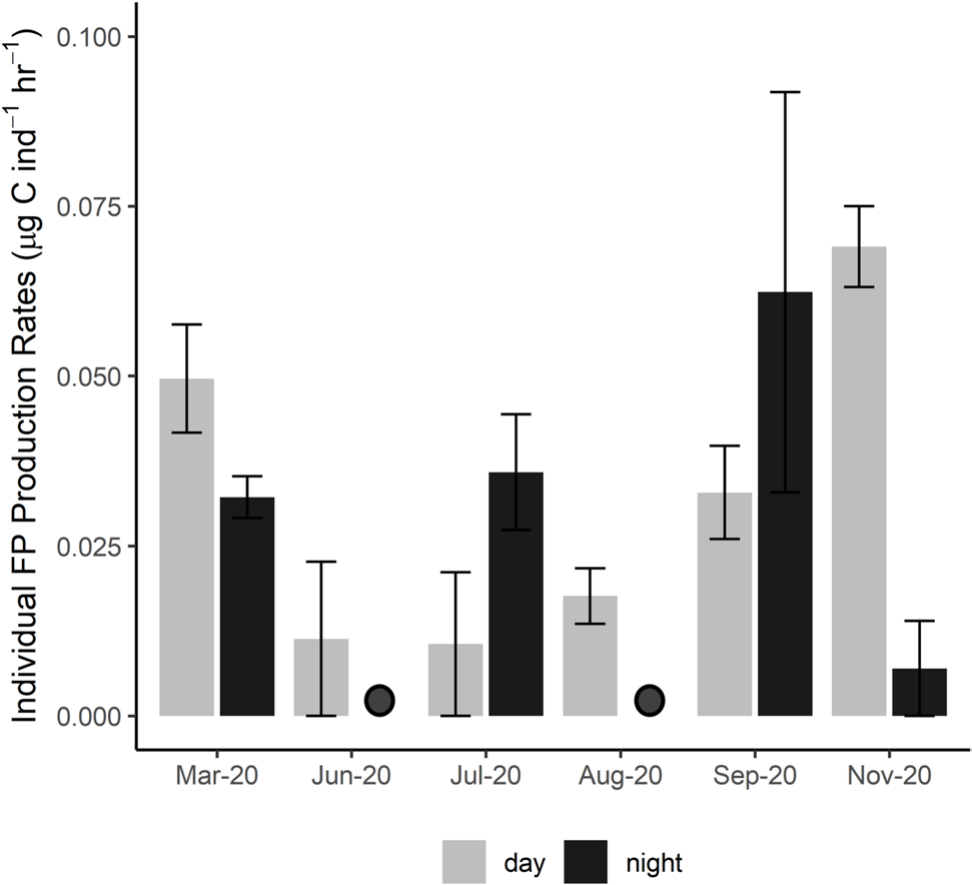
Individual fecal pellet production rates for *Acartia* spp. copepods in the polyhaline York River. Mean rates for daytime (*n* = 6) and nighttime (*n* = 4) experiments are shown; error bars are standard errors among replicates within each experiment. Circles denote nights in which experiments were not performed

Due to diel changes in abundance, individual taxon-specific fecal pellet production experiments with *Acartia* spp. were performed during both day and night while experiments with mysids and isopods were performed only at night (Fig. 5, Table 1). Mysids (*Neomysis americana*) had the highest mean fecal pellet volume (3.07 × 10^−3^ mm^3^ pellet^−1^), *Acartia* spp. pellets were intermediate (1.25 × 10^−3^ mm^3^ pellet^−1^; mean of day and night), and isopods (*Livoneca redmanii*) had the lowest mean fecal pellet volume (4.81 × 10^−4^ mm^3^ pellet^−1^). The mean weight of carbon per fecal pellet was highest in isopods (0.67 µg C pellet^−1^), followed by mysids (0.52 µg C pellet^−1^), and lowest for *Acartia* spp. (0.29 µg C pellet^−1^). The mean fecal pellet carbon to biovolume ratio was highest for isopods (1.21 mg C mm^−3^), followed by *Acartia* spp. (0.55 mg C mm^−3^), and lowest for mysids (0.42 mg C mm^−3^) (Table 1).

**Table 1:** Summary table of results of individual taxon-specific fecal pellet production experiments. Values are mean ± standard error (SE). *n* = number of experiments. For *Acartia* spp., “All” is day and night experiments combined

Taxon	*n*	Day/night	FP volume (mm3 pellet−1)	FPC vol^−1^ (mg C mm−3)	C FP^−1^ (µg C pellet1)	FPC production rate (µg C ind−1 h1)	Weight-specific FPC production rate (µg C mgDW−1 h−1)
*Acartia* spp.	6	Day	**1.40 × 10**^**−3**^** ± **2.68 × 10^−4^	**0.32** ± 0.08	**0.24** ± 0.07	**0.04** ± 0.01	**4.05** ± 1.22
*Acartia* spp.	4	Night	**1.00 × 10**^**−3**^ ± 3.31 × 10^−4^	**0.92** ± 0.29	**0.36** ± 0.06	**0.03** ± 0.01	**3.10** ± 0.86
*Acartia* spp.	10	All	**1.25 × 10**^**−3**^ ± 2.07 × 10^−4^	**0.55** ± 0.13	**0.29** ± 0.04	**0.04** ± 0.01	**3.85** ± 0.37
Mysidacea (*Neomysis americana*)	2	Night	**2.98 × 10**^**−3**^ ± 1.62 × 10^−3^	**0.42** ± 0.21	**0.52** ± 0.10	**0.43** ± 0.09	**0.44** ± 0.25
Isopoda (*Livoneca redmanii*)	2	Night	**4.79 × 10**^**−4**^ ± 8.38 × 10^−5^	**1.21** ± 0.56	**0.67** ± 0.42	**0.24** ± 0.10	**0.01** ± 0.003

The mean *Acartia* spp. individual FPC production rate (0.04 µg C ind^−1^ h^−1^) was an order of magnitude lower than that of mysids (0.43 µg C ind^−1^ h^−1^) and isopods (0.24 µg C ind^−1^ h^−1^) (one-way ANOVA, *p* < 0.0001, df = 39), while rates were more similar between mysids and isopods (paired sample *t*-test, *p* = 0.07, df = 10). Conversely, mean weight-specific FPC production was highest in *Acartia* spp. (3.85 µg C mgDW^−1^ h^−1^), followed by mysids (0.44 µg C mgDW^−1^ h^−1^), and lowest for isopods (0.01 µg C mgDW^−1^ h^−1^) (Table 1). *Acartia* spp. individual FPC production rates were highest in Sept. and Nov. 2020 (Fig. 5). There was no difference in *Acartia* spp. individual FPC production rates between the day and night (paired sample *t*-test, *p* = 0.44, df = 20) (Fig. 5).

### Community-Level Fecal Pellet Production Experiments

**Fig. 6:**
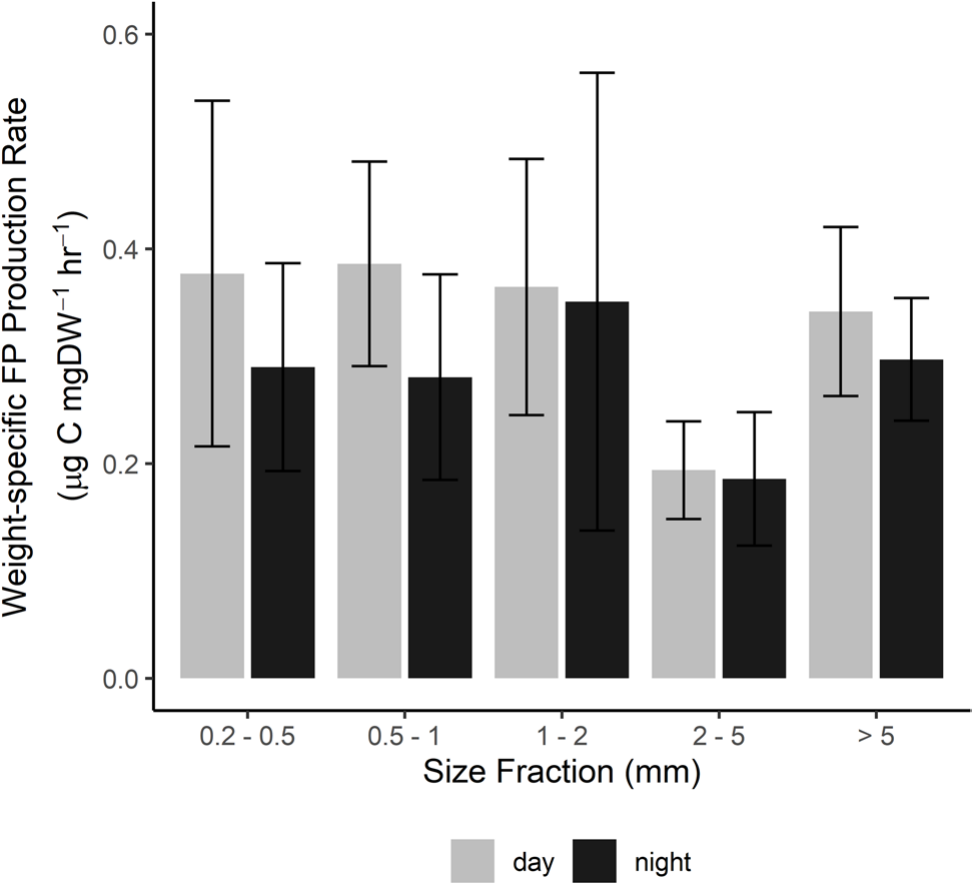
Weight-specific fecal pellet carbon production rates of five mesozooplankton size classes averaged across all experiments in both polyhaline and mesohaline sites in the York River. Mean rates for daytime and nighttime experiments are shown separately; error bars are the standard error of the means (*n* = 11 for day and night)

Weight-specific FPC production rates (mg C mgDW^−1^ h^−1^) were calculated for each size class in each experiment and then averaged across all experiments to provide average rates per size class. Weight-specific rates between the sites were similar (one-way ANOVA, *p* = 0.56, df = 21); thus, results from mesohaline and polyhaline experiments were combined to calculate overall average rates of production per size class (Fig. 6). Generally, fecal pellet volume increased with increasing size class (Table 2, one-way ANOVA, *p* = 0.65, df = 65). Neither volume-specific FPC content nor mean weight-specific FPC production rates differed between size classes (one-way ANOVA, *p* = 0.20 and 0.47, respectively; df = 71 and 109, respectively) (Fig. 6).

**Table 2:** Summary table of results of community-level fecal pellet production experiments. Values are mean ± standard error (SE). n = number of experiments. Mean C FP^−1^ was calculated from mean FP volume and mean FPC vol^−1^ when a direct measurement was not possible

Size fraction	FP volume (mm −3 pellet −1 )	FPC vol^−1^ (mg C mm −3 )	C FP^−1^ (µg C pellet −1 )	FPC production rate (mg C mgDW −1 h −1 )
0.2–0.5 mm	**4.44 × 10**^**−4**^ ± 7.42 × 10^−5^ (*n* = 22)	**0.29** ± 0.11 (*n* = 17)	**8.33 × 10**^**−5**^ ± 2.92 × 10^−5^ (*n* = 22)	**0.35** ± 0.11 (*n* = 11)
0.5–1 mm	**5.90 × 10**^**−4**^ ± 8.08 × 10^−5^ (*n* = 22)	**0.14** ± 0.02 (*n* = 9)	**7.41 × 10**^**−5**^ ± 1.01 × 10^−5^ (*n* = 18)	**0.35** ± 0.07 (*n* = 11)
1–2 mm	**8.32 × 10**^**−4**^ ± 1.58 × 10^−4^ (*n* = 22)	**0.17** ± 0.08 (*n* = 13)	**1.59 × 10**^**−4**^ ± 5.49 × 10^−5^ (*n* = 21)	**0.36** ± 0.10 (*n* = 11)
2–5 mm	**8.89 × 10**^**−4**^ ± 1.59 × 10^−4^ (*n* = 22)	**0.14** ± 0.07 (*n* = 9)	**1.02 × 10**^**−4**^ ± 1.95 × 10^−5^ (*n* = 19)	**0.19** ± 0.04 (*n* = 11)
> 5 mm	**8.74 × 10**^**−4**^ ± 1.16 × 10^−4^ (*n* = 22)	**0.29** ± 0.10 (*n* = 8)	**4.52 × 10**^**−4**^ ± 1.01 × 10^−4^ (*n* = 17)	**0.33** ± 0.05 (*n* = 11)

**Fig. 7:**
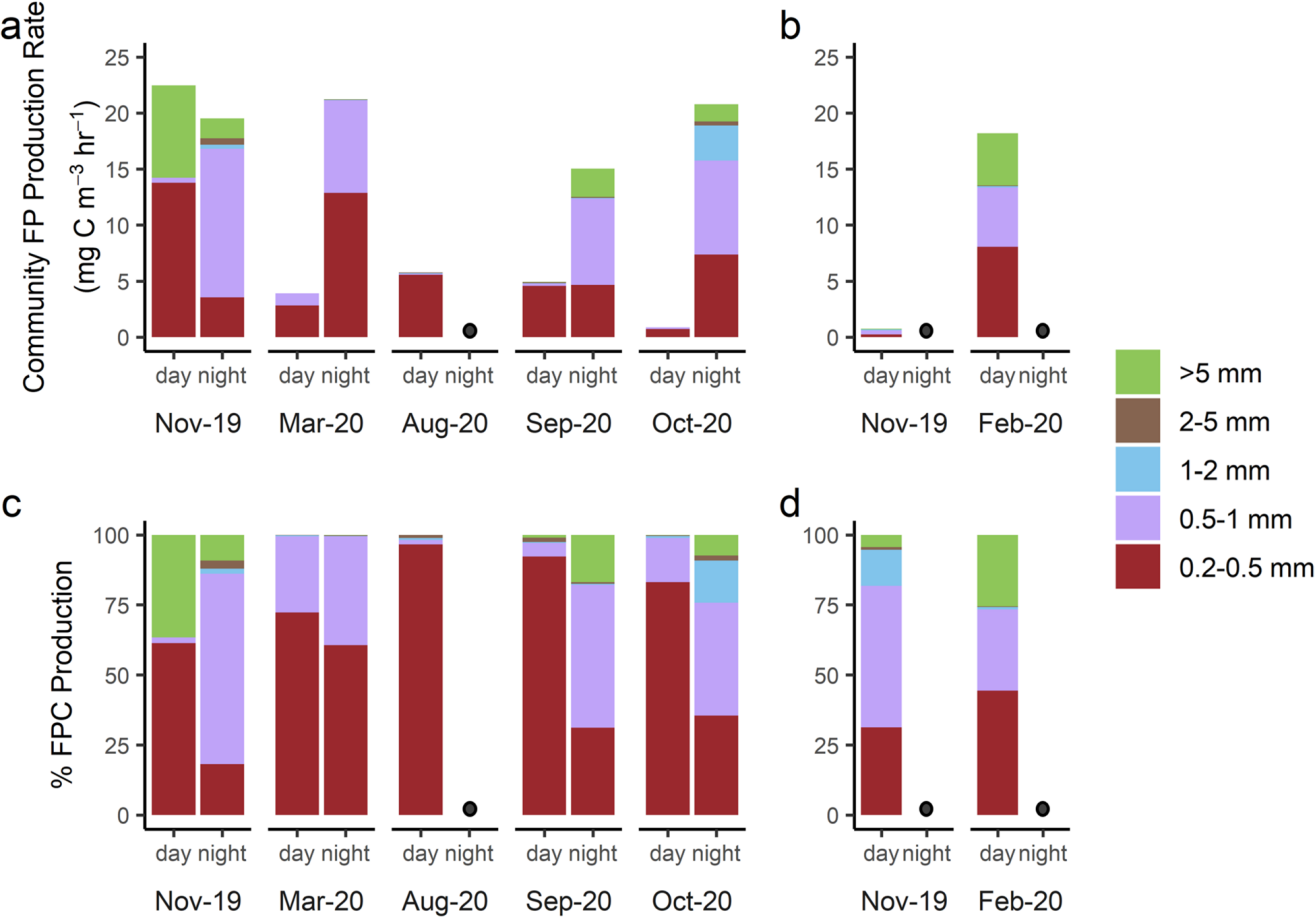
Community fecal pellet carbon production. Biomass-corrected (whole community) fecal pellet carbon production rate for five mesozooplankton size classes in the **a** polyhaline and **b** mesohaline York River, and relative (%) contributions of each size class to total community fecal pellet carbon production in the **c** polyhaline and **d** mesohaline York River. The first bar in the pair is day, and the second is night. Dark circles denote nights in which experiments were not performed

When weight-specific rates were applied to biomass measurements to calculate whole-community FPC production (mg C m^−3^ h^−1^), differences in mean FPC production rates between size classes were observed (one-way ANOVA, *p* = 1.52 × 10^−5^, df = 109) as well as between day and night in the polyhaline site (one-way ANOVA, *p* = 0.02, df = 89), due to differences in biomass. Whole-community FPC production rates were highest in the two smallest size classes in both sites (Fig. 7a, b). Daytime FPC production was highest in Nov. 2019 in the polyhaline site and in Feb. 2020 in the mesohaline site (Fig. 7a and b, respectively). Nighttime FPC production in the polyhaline site was lowest in Sept. 2020 (Fig. 7a).

**Fig. 8:**
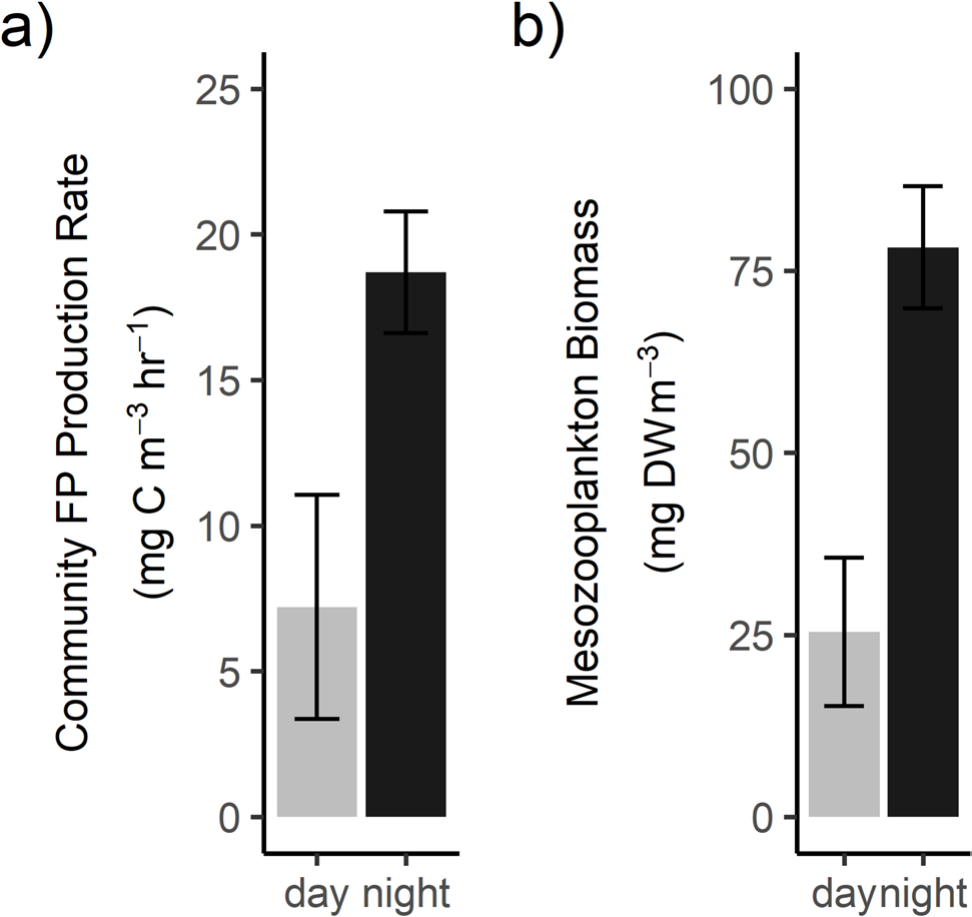
Diel changes in zooplankton fecal pellet carbon production and biomass in the polyhaline York River. **a** Mean whole-community fecal pellet carbon production rates and **b** mean mesozooplankton biomass, in the day (*n* = 5) versus night (*n* = 4). Error bars are standard errors of the means

In the polyhaline site, the smallest size class (0.2–0.5 mm) dominated daytime FPC production across seasons — contributing 62–96% of community FPC production (Fig. 7c). There was a higher contribution of larger size classes to total FPC production at night, with the 0.5–1 mm size class contributing 40–70% of total FPC production at night versus 2–26% during the day (Fig. 7c). In the mesohaline site, the relative contribution of the largest size class (> 5 mm) to total FPC production during the day varied from 4 to 26% (Fig. 7d). Overall, hourly community FPC production rates in the polyhaline site were higher at night (mean 18.7 ± 2.09 mg C m^−3^ h^−1^) than during the day (mean 7.16 ± 3.85 mg C m^−3^ h^−1^) (Fig. 8a), driven by increases in biomass at night due to diel vertical migration (Fig. 8b).

### Seasonal Differences in Fecal Pellet Carbon Production

Using FPC production rates from experiments in 2020 spanning several seasons along with monthly size-fractionated biomass measurements, we estimated seasonal differences in community FPC production at the polyhaline site. We split the sampling periods into three seasons: spring (Feb.–Apr.), summer (Jun.–Sept.), and fall (Oct.–Nov.), calculated the average daytime and nighttime biomass in each season, and applied weight-specific rates from community experiments to calculate daily rates of FPC production per size class for each season, which was integrated over the annual average depth of the euphotic zone for the York River (3.1 m; Schultz, Jr. and Ducklow 2000); we also took into account the changing photoperiod (daylength). Daily community FPC production rates were highest in fall (866 mg C m^−2^ day^−1^), intermediate in spring (444 mg C m^−2^ day^−1^), and lowest in summer (301 mg C m^−2^ day^−1^). Spring FPC production was dominated by the smallest, 0.2–0.5 mm size class (80% of total), while larger size classes contributed more to summer and fall FPC production. In the summer, the largest size class (> 5 mm) contributed 45% of daily FPC production due to high nighttime abundances of large animals (including mysids, chaetognaths, isopods, and hydrozoans). In the fall, the 1–2 mm size class contributed 50% of daily FPC production, driven by increases in the abundance of large non-*Acartia* calanoid copepods (*Pseudodiaptomus* spp. and *Centropages* spp.).

## Discussion

### Diel and Seasonal Trends in Zooplankton Community Structure

Of the 18 major taxa identified in samples, 15 had higher mean density in surface waters at night than during the day. *Acartia* spp. had higher densities on the surface at night, consistent with previous studies of diel vertical migration in Chesapeake Bay tributaries (Cuker and Watson 2002) and other coastal regions (Bollens et al. 1992; Holliland et al. 2012). Additional strong migrators included larval fishes, chaetognaths, and non-*Acartia* spp. calanoid copepods. Larval fishes (e.g., *Anchoa mitchilli*) may benefit from diel vertical migration through up-estuary transport and retention of larvae in areas of high food abundance during times when diel migration patterns are favorably matched with the tidal cycle (North and Houde 2004). Chaetognaths (*Sagitta* spp.) vertically migrate in a variety of ecosystems (Kehayias and Kourouvakalis 2010; Parra et al. 2019), matching vertical distribution with that of their prey (copepods, larval fishes, and decapods) (Kehayias and Ntakou 2008; Steinberg and Condon 2009). Mysids, which we generally only observed at night, are strong diel vertical migrators in the Chesapeake Bay and other estuaries (Calliari et al. 2001; Cuker and Watson 2002) and are disproportionately important in the diets of zooplanktivorous and juvenile demersal fishes in Chesapeake Bay (Sweetman 2018). Barnacle nauplii and cladocerans (predominately *Podon polyphemoides*) were more abundant in surface waters during the day than at night, displaying patterns of reverse diel vertical migration consistent with previous studies (Bosch and Taylor 1973; Cuker and Watson 2002; Valentin et al. 2003). Reverse diel vertical migration reduces predation pressure and resource competition with larger upward-migrating zooplankton (Bollens et al. 1992; Heywood 1996) and enhances retention within the estuary by taking advantage of deep, landward advective currents at night that counteract seaward surface current movement during the day (Bosch and Taylor 1973).

The zooplankton community in the York River was generally dominated year-round (2- to 5-fold above other taxa) by *Acartia* copepods (*A. tonsa* and *A. hudsonica*), consistent with previous studies in the Chesapeake Bay and its tributaries (Price 1986; Cuker and Watson 2002; Kimmel et al. 2012). In addition to *Acartia* spp., the winter and spring zooplankton community included high abundances of the cladoceran *Podon polyphemoides* and barnacle nauplii. Though our data indicate a spring peak in *P. polyphemoides*, long-term Chesapeake Bay zooplankton monitoring data suggest that *P. polyphemoides* typically peaks in July in the mouth of the York River (Steinberg and Condon 2009). Annelids (mostly Spionid larvae) were abundant in the winter in the mesohaline site, and spring in the polyhaline site where they remained abundant through early fall. The increase in non-*Acartia* calanoid copepod densities in February in the mesohaline site was due to *Eurytemora affinis*, which has been observed to peak in abundance in the Chesapeake Bay in March/April (Kimmel and Roman 2004; Steinberg and Condon 2009). Mysids, isopods, larvaceans, and chaetognaths were abundant in the summer and fall in the polyhaline site, and larval meroplankton (decapods, phoronids, fishes, and mollusks) were also abundant in the summer, corresponding with the reported spawning season of many of these taxa from late spring through early fall (Sandifer 1973; Grant and Olney 1983; Marshall and Alden 1985). Non-*Acartia* calanoid copepod (*Centropages* spp. and *Pseudodiaptomus* spp.) abundances increased in September, which is a typical seasonal pattern (Price 1986). Gelatinous zooplankton peaked each summer, their typical “bloom” period in the York River (Condon and Steinberg 2008) and the Chesapeake Bay more broadly (Purcell et al. 1994; Stone et al. 2019). Ctenophores were most abundant in Nov. 2019 with a secondary peak in summer 2020, which is consistent with long-term observations of ctenophore abundance in the lower Bay that indicate a peak in June/July (Stone et al. 2019). Hydromedusae (especially *Nemopsis bachei*) were abundant throughout the sampling period and are key predators of *A. tonsa* copepodites and nauplii throughout the fall in the southern Chesapeake Bay, thus competing for food with larval fishes and influencing fish recruitment (Purcell et al. 1999).

### The Importance of Small-Size Classes, Including *Acartia* spp. Copepods, to Community Fecal Pellet Carbon Production

Weight-specific FPC production did not strongly differ between size classes in community-level experiments, and therefore, the relative contribution of each size class to total FPC production was driven by their biomass. Thus, community FPC production was dominated by the smallest size class (0.2–0.5 mm) during daytime experiments with increasing relative contributions by larger size classes (particularly the 0.5–1 mm size class) at night, due to diel vertical migration of larger animals to the surface. The 0.2–0.5 and 0.5–1 mm size classes were mainly composed of *Acartia* spp. which dominated the zooplankton community, constituting up to 99% of total animal abundance.

The average carbon content of fecal pellets produced by *Acartia* spp. (0.29 µg C pellet^−1^) in our individual species FPC production experiments was higher than in a previous study in the York River (0.02 µg C pellet^−1^; Saba et al. 2011), but within the range of pellet carbon contents of *A. tonsa* feeding on large diatoms under simulated phytoplankton bloom conditions (0.03–0.38 µg C pellet^−1^; Butler and Dam 1994) and of *A. hudsonica* feeding on coccolithophores (0.13–0.28 µg C pellet^−1^; Honjo and Roman 1978). The individual fecal pellet production rate of *Acartia* spp. in our study (0.25 pellets ind^−1^ h^−1^) is lower than previous studies of *A. tonsa* feeding on large diatoms in the York River (2.8 pellets ind^−1^ h^−1^; Saba et al. 2011) and mixed calanoid copepod (including *Acartia* spp.) fecal pellet production rates in the Yangtze estuary in summer (0.62–1.34 pellets ind^−1^ h^−1^; Guo and Sun 2018). Our mean per-individual pellet production rates may be comparatively low due to differences in phytoplankton community structure or experimental artifacts such as the settling of large diatoms causing food limitation in the jars or the addition of small animals in incubation seawater that may have consumed phytoplankton or fecal pellets. Our calculated mean individual FPC production rate for *Acartia* spp. (0.04 µg C ind^−1^ h^−1^) was similar to another coastal calanoid copepod, *Temora longicornis* (0.05–0.07 µg C ind^−1^ h^−1^; Ploug et al. 2008). Weight-specific FPC production rate for *Acartia* spp. (mean = 3.9 µg C mgDW^−1^ h^−1^) was nearly fourfold higher than for the small calanoid copepod *Clausocalanus lividus* (1.1 µg C mgDW^−1^ h^−1^) in the Northeast Pacific Ocean (Stamieszkin et al. 2021), where iron limitation limits primary production and consequently higher trophic levels (secondary production).

The proportionately high contribution of < 1 mm size classes to overall community fecal pellet production is consistent with fecal pellet production studies in other estuaries and in the open ocean. Zooplankton in the 0.5–1 mm size class (dominated by calanoid copepods) produced over 50% of all fecal pellets in the highly productive Changjiang (Yangtze) estuary (Guo and Sun 2018). In the subarctic Northeast Pacific Ocean, Stamieszkin et al. (2021) also show a high contribution of small size classes to total community FPC production; while the smallest size class (0.2–0.5 mm) contributed just 0.2–3% to total biomass, it contributed the most (32%) to total community FPC production. In our study, although *Acartia* spp. had significantly lower individual FPC production rates than larger animals (mysids, isopods), high *Acartia* spp. density in the estuary leads to its dominance in community FPC production.

Large animals were likely underrepresented in community FPC production experiments due to their relative rareness, as well as evasion (due to faster swimming compared to smaller taxa) during subsampling for experiments that would have excluded them from incubations. Taxon-specific FPC production experiments revealed that while larger animals (mysids and isopods) had lower weight-specific FPC production rates than *Acartia* spp., they had higher individual rates of FPC production (pellets ind^−1^ h^−1^) and created larger, more carbon-rich pellets. The contribution of these larger taxa to total community FPC production was thus likely underestimated, and care should be taken to target and include these animals in future experiments and estimates of community-level FPC production.

### Diel Differences in Fecal Pellet Carbon Production

Community FPC production rates were significantly higher (mean = 19-fold, range = 3- to 65-fold) in surface waters at night than during the day, driven by increases in biomass at night due to diel vertical migration of larger animals into surface waters. This finding is consistent with the results of Stamieszkin et al. (2021) in the subarctic Northeast Pacific Ocean where FPC production at night was on average threefold that during the day. In our study, the biomass of the 0.5–1 mm and 1–2 mm size classes increased the most on average from day to night, due to the migration of copepods, such as *Acartia* spp. and the larger *Centropages* spp. and *Pseudodiaptomus* spp., as well as chaetognaths, larval decapods, and fishes. *Acartia* spp. FPC production rate per individual was not significantly different between day and night, implying that feeding rates did not increase at night. Thus, increases in the abundance of *Acartia* spp. and other calanoid copepods on the surface at night drove the large increase in community FPC production from day to night. Biomass of the largest size class (> 5 mm) increased substantially between the day and night in the summer and fall due to the presence of mysids, which occurred in surface waters only at night. While mysids had relatively high FPC production rates, their abundance was relatively low compared to that of smaller animals such as copepods. The biomass of the > 5 mm size class also increased between day and night due to the presence of large scyphozoans such as *Chrysaora chesapeakei* and *Aurelia aurita*. *Chrysaora* medusae show negative phototaxis (movement away from a directional light source) in both natural and manipulated light conditions in mesocosms (Schuyler and Sullivan 1997), and *Aurelia aurita* vertically migrate into surface waters at dusk where they can produce swarms (Malej et al. 2007). Scyphomedusae were not included in fecal pellet production experiments due to their large size but may exert top-down control of FPC flux in the Chesapeake Bay via trophic cascade (Stone and Steinberg 2018; described below).

Diel differences in FPC production rates may have been affected by coprophagy (ingestion of fecal pellets) and coprorhexy (physical fragmentation of fecal pellets) by small (< 200 µm), seasonally abundant cladocerans and barnacle larvae, which were often found in the contents of the outer jars of fecal pellet production experiments along with filtered fecal pellets. Zooplankton in this size class play an important role in coprophagy and restricting vertical fecal pellet carbon flux (Poulsen and Kiørboe 2006); thus, their relatively high abundance during the day may have led to higher rates of coprophagy and coprorhexy in daytime fecal pellet production experiments, contributing to the observed increase in community FPC production rates from day to night.

### Seasonal Differences in Fecal Pellet Carbon Production

Community FPC production was highest in fall due to increased diversity and abundance of larger zooplankton and lowest in summer likely due to top-down control of abundant crustacean taxa by gelatinous predators. In the fall, there was a comparatively higher abundance and diversity of relatively larger animals, including non-*Acartia* copepods such as *Centropages* and *Pseudodiaptomus*, mysids, isopods, chaetognaths, and larval meroplankton. This higher abundance of larger animals, producing larger fecal pellets with high carbon content, likely contributed to overall higher fall community FPC production. The comparatively lower summer community FPC production may be due to the presence of gelatinous zooplankton, which can exert seasonal top-down control on FPC production through cascading trophic effects of the scyphozoan *Chrysaora chesapeakei* and ctenophore *Mnemiopsis leidyi* on copepods including *Acartia tonsa* (Stone and Steinberg 2018). In mesocosm experiments in the York River, the presence of *M. leidyi* reduced their prey copepod densities, leading to a 50% decrease in copepod FPC flux (from 36 to 18 µg C m^−3^ day^−1^ without and with *M. leidyi*, respectively; Stone and Steinberg 2018). However, *C. chesapeakei* preys on *M. leidyi*, thus when present in large numbers can reduce predation pressure on copepods and lead to increased copepod FPC production and flux. The relatively high abundance of ctenophores and hydrozoans, which are efficient predators of larval mesozooplankton including copepodites and barnacle nauplii (Purcell and Nemazie 1992), in September 2020 may be partially responsible for lower rates of FPC production in the polyhaline site in the summer versus fall and spring. In addition, while community FPC production experiments sometimes included ctenophores (*M. leidyi* and *Beroë ovata*), it is difficult to identify the mucous masses egested after their feeding (versus “proper” fecal pellets produced by other taxa); thus, ctenophore fecal production is excluded in our experiments. Mesocosm experiments with ctenophores also show that clearance rates increase with increasing mesocosm size (Purcell and Cowan Jr. 1995); thus, the 4-l incubation containers used in our experiments may not have been of sufficient volume for ctenophores to clear copepod prey, also leading to underestimation of the role of ctenophores in FPC production in the summer and fall. Finally, while increases in individual FPC production in summer may be expected due to increased metabolism in warmer temperatures, we posit that at the community level, reduced abundance of crustacean zooplankton by gelatinous zooplankton predation counteracts any seasonal changes in metabolism.

### Implications for Fecal Pellet Carbon Vertical Export in the York River

This study analyzed patterns of zooplankton FPC production, not the fate (vertical export) of this FPC, which would take into consideration factors that affect the attenuation of sinking particles. FPC production thus represents the maximum possible POC that could reach the benthos through FPC export. Applying average photoperiod (day/night lengths) to our data and integrating over the average depth of the euphotic zone (3.1 m; Schultz, Jr. and Ducklow, 2000), our estimate of FPC production in polyhaline York River surface waters (mean 928 mg C m^−2^ day^−1^, range 699–1158 mg C m^−2^ day^−1^) is a maximum estimate of potential flux if no pellets are attenuated within the water column. For comparison, in the Yangtze estuary mean potential FPC flux from mixed copepods ranged from 34.6 to 64.4 mg C m^−2^ day^−1^ in the spring and 51.8–89.0 mg C m^−2^ day^−1^ in the summer (Guo and Sun 2018). Their study was based on vertical net tows performed from 5 m above the sediment floor to the surface, and the FPC values are substantially lower than in our study, likely due to the exclusion of non-copepod taxa and migrators, including copepods, that spend daylight hours within or near the sediment floor. Further, our estimate of community FPC production is two orders of magnitude higher than in a study using the same methods in the subarctic Northeast Pacific Ocean (mean 3.1 mg C m^−2^ day^−1^), which represents a low flux endmember of the biological pump, being an open ocean, high nutrient-low chlorophyll (HNLC) region (Stamieszkin et al. 2021). In the Northeast Pacific Ocean, the average biomass of zooplankton in the euphotic zone was roughly half of the biomass in the euphotic zone in our study. Thus, the higher rates of community FPC production in our study can be attributed to higher zooplankton biomass, as well as low fecal pellet production rates by the dominant copepod, *Neocalanus* spp., in the Northeast Pacific (Stamieszkin et al. 2021).

Factors that control the attenuation or fate of zooplankton fecal pellets produced in surface waters include pellet sinking rate variability, coprophagy and coprophexy, and bacterial remineralization (Lampitt et al. 1990; Poulsen and Kiørboe 2006; Stukel et al. 2011). Coprophagy of fecal pellets by zooplankton plays a particularly important role in the attenuation of fecal pellets in regions dominated by small copepods, such as the strait of Øresund between Denmark and Sweden, where most pellets produced on the surface were attenuated within the upper 50 m of the water column (Poulsen and Kiørboe 2006). In the subarctic Northeast Pacific, sediment trap analysis revealed that fecal pellets egested from small mesozooplankton are highly abundant within the upper epipelagic zone but are attenuated rapidly with depth (on average 86% were attenuated by 100 m), therefore contributing little to total POC flux to the mesopelagic zone (Stamieszkin et al. 2021; Durkin et al. 2021). Sediment trap studies in estuaries that quantify vertical FPC export reveal up to 1.3% of total POC in traps at depths of 10–50 m can be attributed to FPC, with the majority of POC being other detritus (Waite et al. 2005; Svensen et al. 2007). The sediment trap depths in these estuarine studies are deeper than the York River — which has main channel depths ranging from 6 to 24 m (Friedrichs 2009); thus, the relative magnitude of FPC export in the York River is likely more tightly coupled to surface FPC production due to less vertical distance for attenuation within the water column.

In estuaries, physical processes such as resuspension and flushing due to river flow and the tidal cycle are also important in the attenuation or fate of fecal pellets. For example, tidally forced sediment resuspension can regularly exceed 1 g L^−1^ at peak tidal flow in the York River (Friedrichs 2009). Sediment trap studies show that tidal resuspension can cause resilient fecal pellets produced by benthic polychaetes (which are of similar size and shape to *Acartia* fecal pellets) to be resuspended throughout the water column, including into the surface 2 m (Massey et al. 2018). Seasonal changes in stratification may also play a role in the fate of FPC. During summer, the York River experiences increased water column stratification and periodic bottom hypoxia due to eutrophication and decomposition of algal cells (Lake et al. 2013). This stratification may prevent the reintroduction of fecal pellets into surface waters by tidal forcing.

Comparison to net primary production (NPP) in the York River provides further context for our results. Our mean estimate for FPC production (0.93 g C m^−2^ day^−1^), and thus potential FPC export, falls mid-range of direct measurements of summer water column NPP (0.43–1.66 g C m^−2^ day^−1^; Lake et al. 2013), exceeds modeled mean seasonal estimates of NPP (0.37 g C m^−2^ day^−1^ in fall and 0.88 g C m^−2^ day^−1^ in spring and summer; Lake and Brush 2015), and is one-quarter of maximum spring NPP (model estimate 3.64 g C m^−2^ day^−1^; Lake and Brush 2015). While ultimately maximum FPC production (and export) cannot exceed NPP (particle production), the time scales of these measurements are different, and this comparison shows potential for significant vertical export of NPP as FPC.

### Conclusions and Future Considerations

Estuaries such as the Chesapeake Bay and its tributaries can surpass oceanic systems in terms of their contribution to FPC production due to relatively high densities of small zooplankton, which were the dominant contributors to community FPC production in our study. While measurements from the Northeast Pacific (with consistent methods) represent a low-flux end member, comparisons between the results of this study and that of the Northeast Pacific show that estuaries can have a higher rate of FPC production and potential vertical export of carbon than open ocean systems, which are more frequently studied regarding their role in the biological carbon pump.

This study focused on the production of zooplankton fecal pellets, not their sinking rates or fate. Understanding the processes that contribute to fecal pellet attenuation in estuaries is critical in determining the role of fecal pellet production in vertical carbon export. Further, this study did not account for the active transport of FPC to depth by diel migrating species (e.g., Schnetzer and Steinberg 2002). Our study suggests that consideration of the diel cycle is critical for understanding diel and seasonal changes in potential FPC flux in estuaries, particularly because some of the most abundant taxa are diel vertical migrators. Sediment trap studies in estuaries that include techniques such as polyacrylamide gel traps for gentle collection of intact particles enabling classification of particle types (e.g., Durkin et al. 2021), as well as discrete multiple-depth, diel sampling of zooplankton, would help to determine the fate of fecal pellets produced in the surface waters and their contribution to estuarine benthic-pelagic coupling.

This study provides a baseline for future analysis of long-term changes in zooplankton community structure and carbon cycling in the Chesapeake Bay region. Beaugrand et al. (2010) showed long-term latitudinal changes in copepod biodiversity and their fecal pellet surface residence time in the North Atlantic, with copepod diversity increasing over time in the northern latitudes due to increasing water temperatures and species range extensions. Copepod body size was negatively correlated with diversity, suggesting an overall decrease in copepod body size leading to smaller, more slowly sinking fecal pellets (Beaugrand et al. 2010). Increased residence time of pellets in the epipelagic zone increases the likelihood of remineralization and decreases the likelihood of carbon burial and sequestration, impacting the ocean carbon cycle and our climate system. Estuaries are expected to be similarly impacted by climate change (Irby et al. 2018), with projected increases in water temperature over time, but also sea level rise causing shifts in the salinity regime and a variety of factors influencing dissolved oxygen concentration throughout the water column. These changes would collectively affect zooplankton horizontal and vertical distribution, as well as survival, and thus zooplankton-mediated carbon cycling. The role of the biological pump in estuaries has rarely been examined but is needed to improve carbon cycling models and to understand the effects of climate change on estuarine ecosystems.

## Supplementary Information

Below is the link to the electronic supplementary material.

Supplementary file1 (DOCX 718 KB)
