# Supplementary material for: The Role of Zooplankton Community Composition in Fecal Pellet Carbon Production in the York River Estuary, Chesapeake Bay
Source: Estuaries Coast. 2024 Nov 13;48(1):17. doi: 10.1007/s12237-024-01442-8 (PMC11561122; doi:10.1007/s12237-024-01442-8)
Supplement: Supplementary file 1 — Supplementary file1 (DOCX 718 KB) [file 12237_2024_1442_MOESM1_ESM.docx]

**Electronic Supplementary Material**

**Title:** The role of zooplankton community composition in fecal pellet carbon production in the York River Estuary, Chesapeake Bay

**Journal:** *Estuaries and Coasts*

**Authors:**

Kristen Sharpe^1^ (corresponding author), [knsharpe@vims.edu](mailto:knsharpe@vims.edu), (804) 684-7069, ORCID ID: 0000-0001-6573-9031

Deborah K. Steinberg^1^, [debbies@vims.edu](mailto:debbies@vims.edu), ORCID ID: 0000-0001-9884-4655

^1^Virginia Institute of Marine Science, 1375 Greate Rd., Gloucester Point, VA, 23062 USA

Karen Stamieszkin^2^, [kstamieszkin@bigelow.org](mailto:kstamieszkin@bigelow.org), ORCID ID: 0000-0001-6336-8740

^2^Bigelow Laboratory for Ocean Sciences, 60 Bigelow Dr., East Boothbay, ME, 04544 USA

**Submission to *Estuaries and Coasts* Date:** November 14, 2023

**Table S1** List of major taxa identified from York River zooplankton samples. Copepoda includes the most abundant calanoid copepod genus, *Acartia* (*A. tonsa* and *A. hudsonica*), all other calanoid copepods combined, and three other orders of copepods. Balanidae includes barnacle larvae. Cnidaria includes two classes: scyphozoans with large free-living medusae such as bay nettles, and relatively smaller (< 10 mm) predatory hydrozoans (nearly exclusively *Nemopsis bachei*). Teleostei includes larval fishes

| **Taxonomic Categories** | **Sub-Categories** |
| --- | --- |
| Copepoda | *Acartia* spp. |
|  | Other Calanoida |
|  | Cyclopoida |
|  | Harpacticoida |
|  | Siphonostomatoida |
| Cladocera |  |
| Balanidae |  |
| Decapoda |  |
| Mysidacea |  |
| Isopoda |  |
| Ctenophora |  |
| Cnidaria | Scyphozoa |
|  | Hydrozoa |
| Mollusca |  |
| Chaetognatha |  |
| Annelida |  |
| Phoronida |  |
| Larvacea |  |
| Teleostei |  |


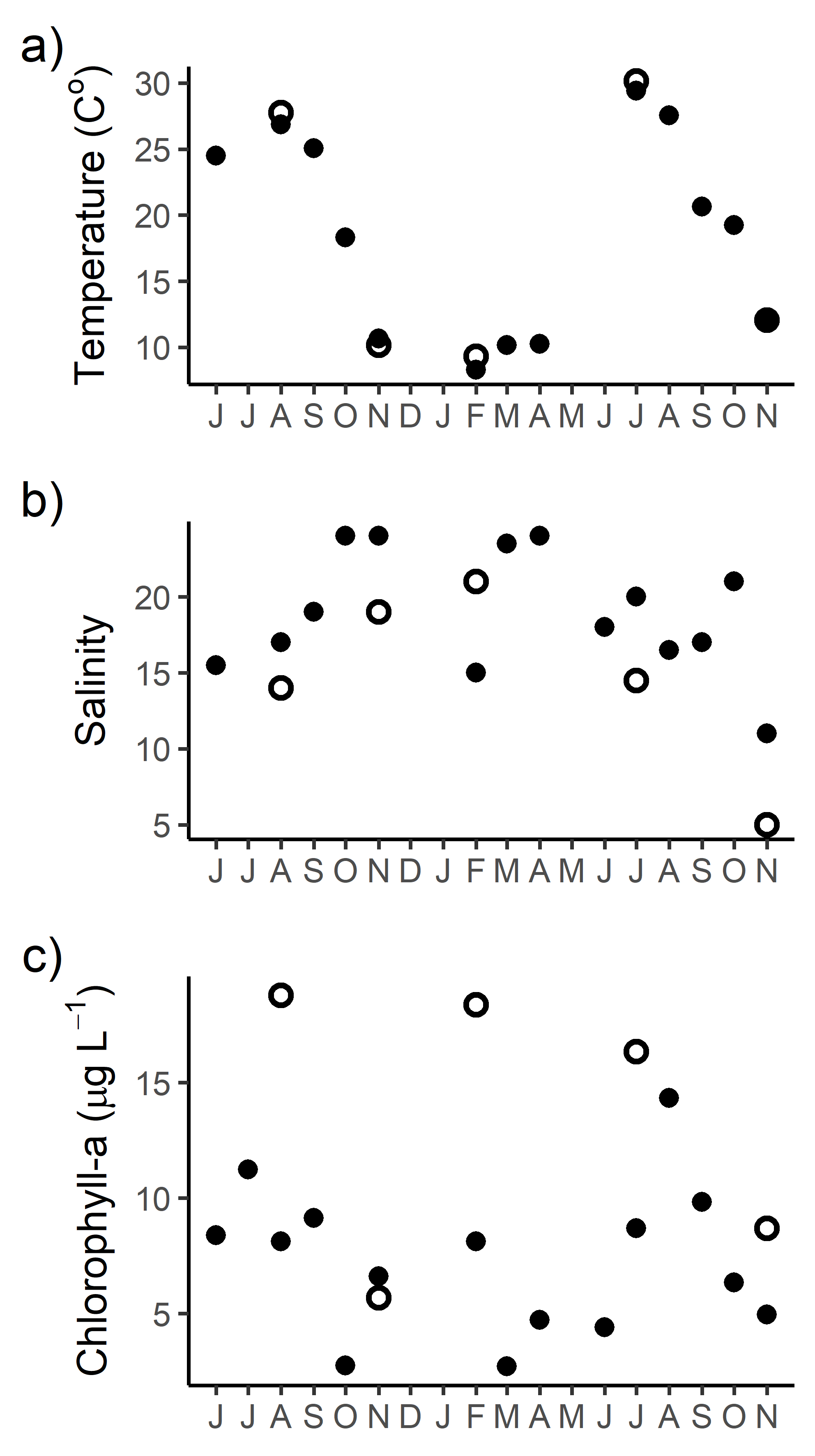


**Fig. S1** Monthly (a) temperature, (b) salinity, and (c) chlorophyll-a concentrations from June 2019 to November 2020 in the polyhaline (open circles) and mesohaline (filled circles) sites in the York River. Values are average of daytime and nighttime measurements

**
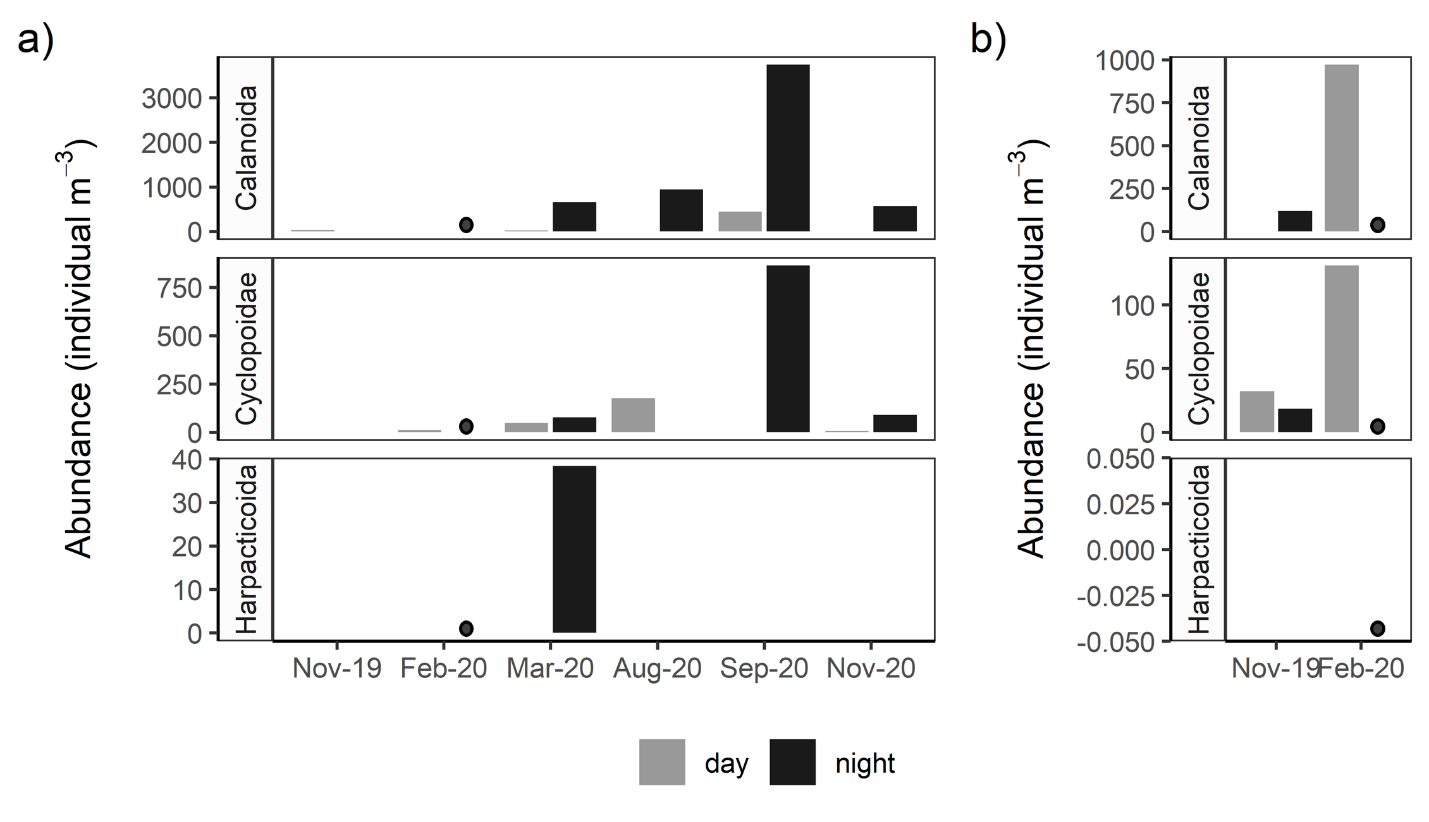
**

**Fig. S2** Monthly densities of the three major taxa of non-*Acartia* copepods in the polyhaline (a) and mesohaline (b) York River during the day and night. The dark circles in February 2020 denote no night sampling performed, to distinguish from absence of taxa in other months. See Table S1 for full list and explanation of major taxonomic categories. For all tows n=1

**
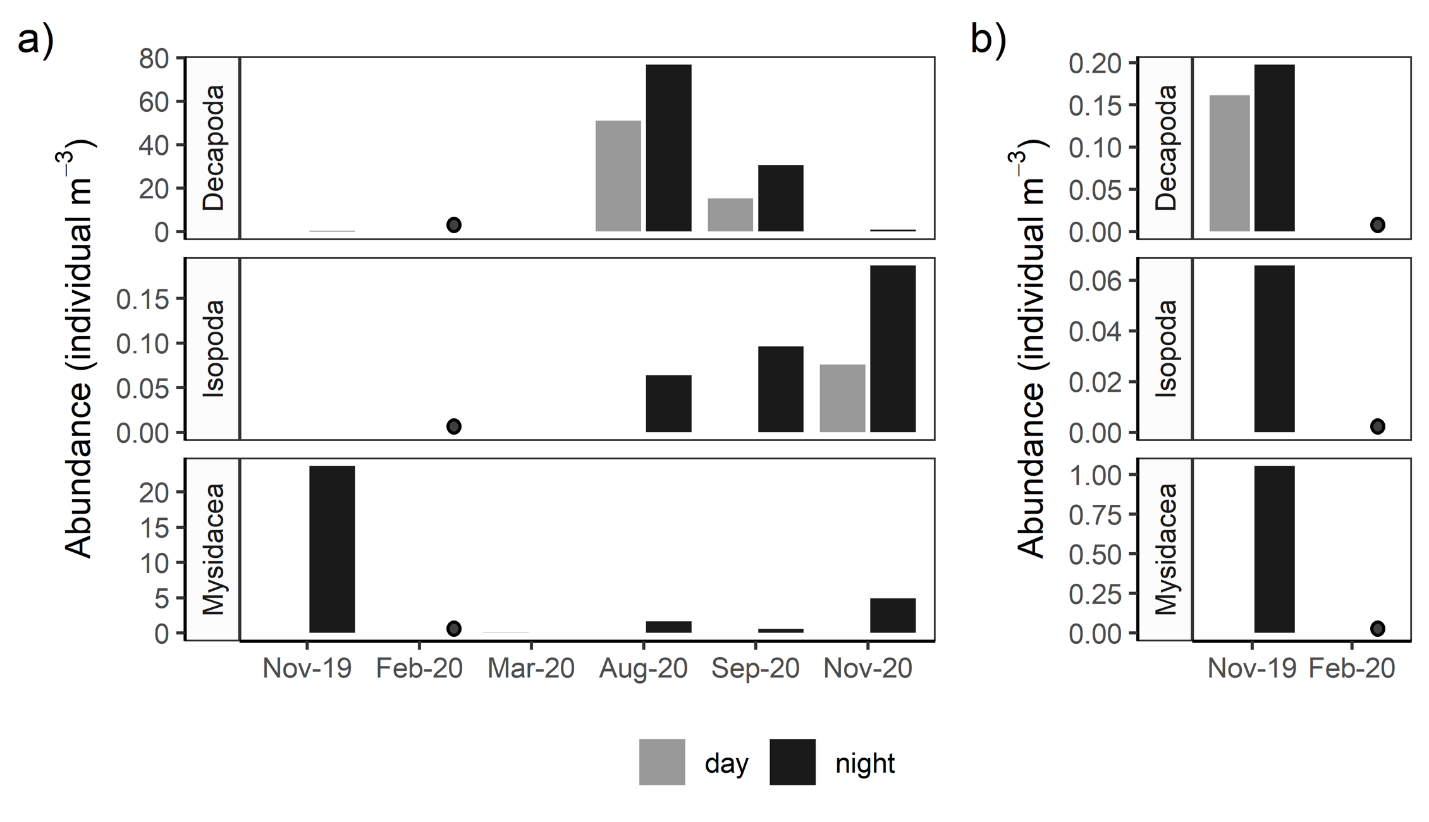
**

**Fig. S3** Monthly densities of three additional major taxa of crustaceans in the polyhaline (a) and mesohaline (b) York River during the day and night. The dark circles in February 2020 denote no night sampling performed, to distinguish from absence of taxa in other months. See Table S1 for full list and explanation of major taxonomic categories. For all tows n=1

**
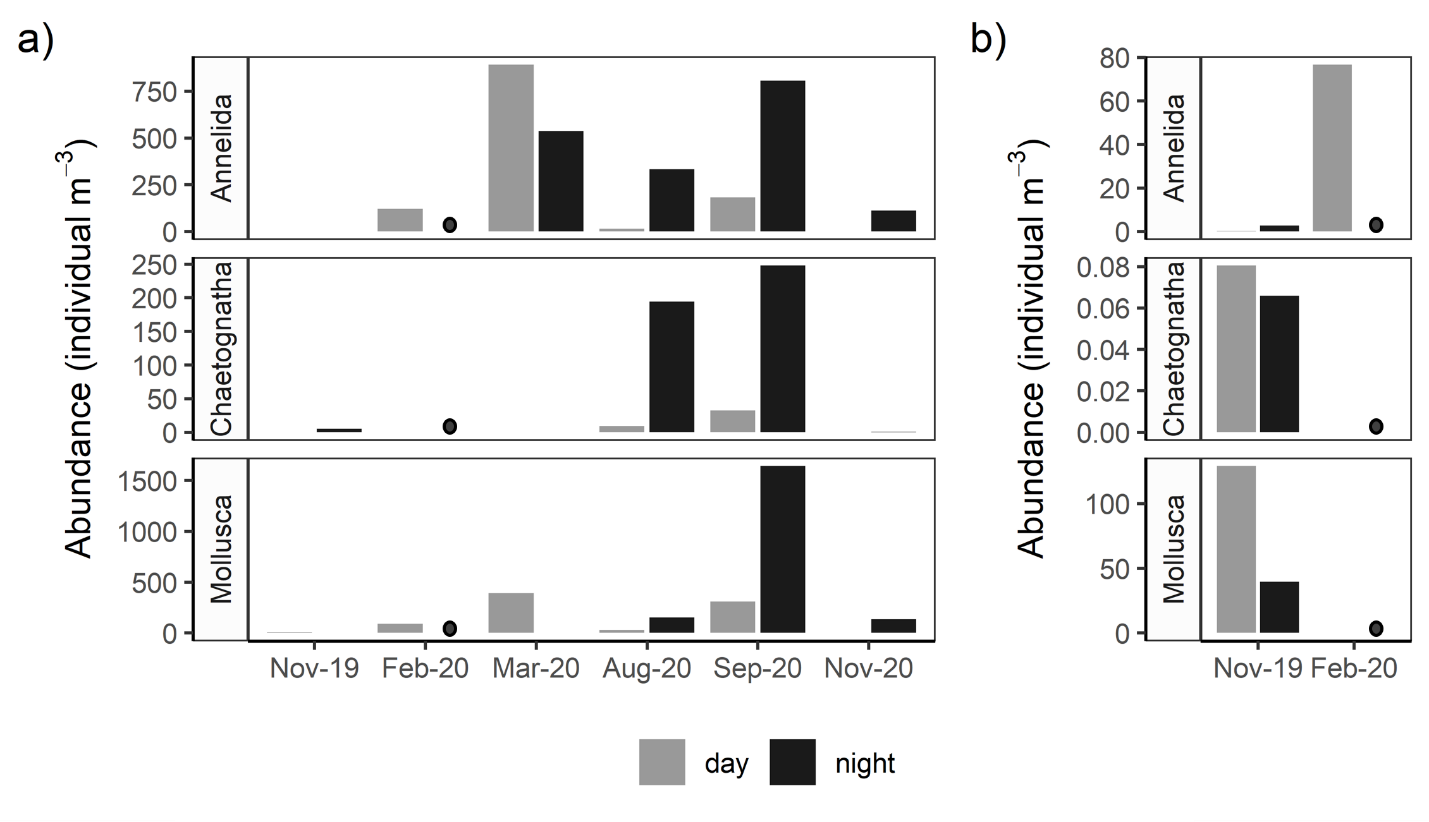
**

**Fig. S4** Monthly densities of abundant non-crustacean zooplankton taxa in the polyhaline (a) and mesohaline (b) York River during the day and night. The dark circles in February 2020 denote no night sampling performed, to distinguish from absence of taxa in other months. See Table S1 for full list and explanation of major taxonomic categories. For all tows n=1

**
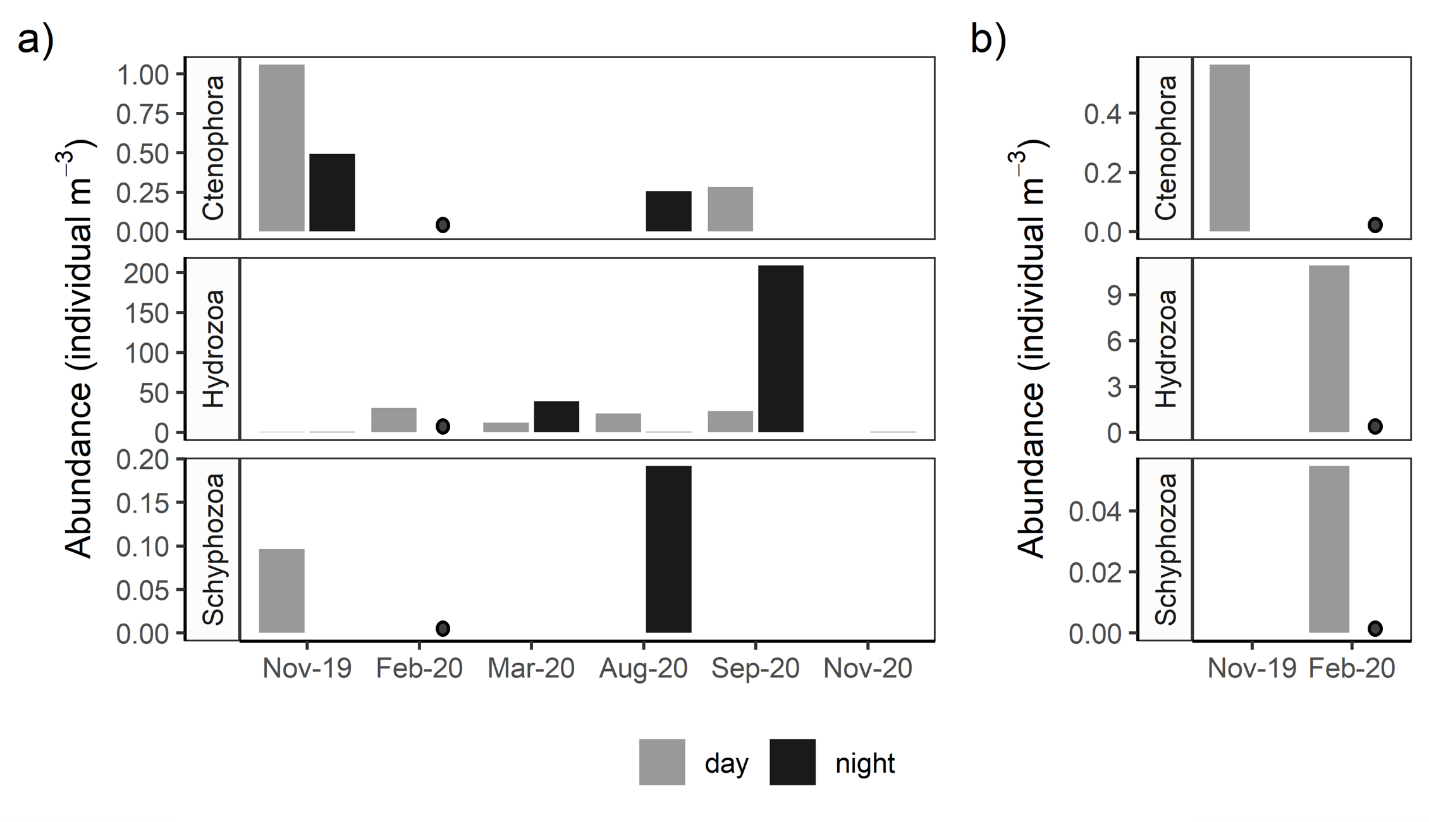
**

**Fig. S5** Monthly densities of the three major taxa of gelatinous zooplankton in the polyhaline (a) and mesohaline (b) York River during the day and night. The dark circles in February 2020 denote no night sampling performed, to distinguish from absence of taxa in other months. See Table 1 for full list and explanation of major taxonomic categories, For all tows n=1
